# Role of the central cations in the mechanical unfolding of DNA and RNA G-quadruplexes

**DOI:** 10.1093/nar/gkv690

**Published:** 2015-07-13

**Authors:** Ana Elisa Bergues-Pupo, J. Ricardo Arias-Gonzalez, María Carmen Morón, Alessandro Fiasconaro, Fernando Falo

**Affiliations:** 1 Departamento de Física de la Materia Condensada, Facultad de Ciencias, Universidad de Zaragoza, Pedro Cerbuna 12, 50009 Zaragoza, Spain; 2 Instituto de Biocomputación y Física de Sistemas Complejos (BIFI), Universidad de Zaragoza, Mariano Esquillor, 50018 Zaragoza, Spain; 3 Departamento de Física, Universidad de Oriente, 90500 Santiago de Cuba, Cuba; 4 Instituto Madrileño de Estudios Avanzados en Nanociencia (IMDEA Nanociencia), Cantoblanco, 28049 Madrid, Spain; 5 CNB-CSIC-IMDEA Nanociencia Associated Unit ‘Unidad de Nanobiotecnología, Madrid, Spain’; 6 Instituto de Ciencia de Materiales de Aragón (ICMA), Consejo Superior de Investigaciones Científicas, Universidad de Zaragoza, Pedro Cerbuna 12, 50009 Zaragoza, Spain; 7 School of Mathematical Sciences, Queen Mary University of London - Mile End Road, London E1 4NS, UK

## Abstract

Cations are known to mediate diverse interactions in nucleic acids duplexes but they are critical in the arrangement of four-stranded structures. Here, we use all-atom molecular dynamics simulations with explicit solvent to analyse the mechanical unfolding of representative intramolecular G-quadruplex structures: a parallel, a hybrid and an antiparallel DNA and a parallel RNA, in the presence of stabilising cations. We confirm the stability of these conformations in the presence of $\rm {K}^+$ central ions and observe distortions from the tetrad topology in their absence. Force-induced unfolding dynamics is then investigated. We show that the unfolding events in the force-extension curves are concomitant to the loss of coordination between the central ions and the guanines of the G-quadruplex. We found lower ruptures forces for the parallel configuration with respect to the antiparallel one, while the behaviour of the force pattern of the parallel RNA appears similar to the parallel DNA. We anticipate that our results will be essential to interpret the fine structure rupture profiles in stretching assays at high resolution and will shed light on the mechanochemical activity of G-quadruplex-binding machinery.

## INTRODUCTION

DNA and RNA G-quadruplexes are important non-canonical, four-stranded structures in which G-rich sequences can self-assemble in regular shapes in the presence of monovalent cations. The basic motif, named the G-tetrad (1,2), is formed via Hoogsteen hydrogen bonding between four guanine nucleobases in a planar arrangement. Several G-tetrads can stack successively on top of each other to form the G-quadruplex. The presence of positive ions like $\rm {K}^+$, $\rm {Na}^+$ and $\rm {NH}_4^+$ inside the structure stabilises the highly electronegative central channel along the axis of the G-quadruplex stem. DNA G-quadruplexes have been observed at telomere ends and in gene promoter regions (3–5). On the other hand, RNA G-quadruplexes form in mRNA or by the transcription of the telomeric DNA into the repeat-containing RNA (TERRA) (6–8). G-quadruplexes seem to play a major structural and regulatory role in the chromosomal maintenance and in the control of gene expression. Also, their ligand binding properties make them excellent targets for therapeutic agents (9–11).

G-quadruplexes can adopt manifold topologies characterised by different orientation of the four strands in the quartet stem. If all the strands point in the same direction, the structure is parallel and otherwise antiparallel. DNA is able to fold into both conformations (12–17) while RNA has been only observed in the parallel one (18–20). Force spectroscopy experiments have shown that the different G-quadruplex conformations exhibit different mechanical stabilities (20–25). For instance, the antiparallel DNA structures have proved to exhibit higher rupture forces than the corresponding parallel ones (22–24). DNA and its RNA counterpart quadruplex have different mechanical stabilities as well (20,25). Understanding the mechanochemistry of those different topologies can provide valuable information concerning the overall stability, flexibility, ligand binding affinity and the folding pathway of the G-quadruplexes.

Of critical importance for the stabilisation and unfolding paths of these key cellular components, not found in DNA or RNA duplexes (26), are the ions within the central channel (27). Their position has been clearly identified in static, structural experiments and in spontaneous folding pathways (28,29), but their importance in the force-induced unfolding dynamics has not been studied in depth.

Molecular dynamics (MD) simulation is a valuable tool that has been widely used to complement experiments and to provide deeper insights in the understanding of different properties of the G-quadruplexes at an atomic level. Most of the MD studies have been conducted at equilibrium conditions in order to check the conformational stability and the dynamics of the G-quadruplexes (30–35). These equilibrium simulations have validated the choice of the AMBER force fields to capture the main characteristics of the G-quadruplex dynamics (32,33). MD have been also carried out to make clear the role of the monovalent cations in stabilising the quartet stems (36–39), features otherwise not revealed by the experimental approaches. They agree that the cations confer a great stability to the G-quadruplex and that removing them leads to a great distortion of the G-quadruplex structure.

Steered Molecular Dynamics (SMD) allows the simulation of the application of external forces to specific atoms, in a similar way to the stretching experiments performed in single molecules by optical and magnetic tweezers. This method provides an alternative path to characterise the mechanical stability of G-quadruplexes by computing the rupture forces and the free energy differences along unfolding coordinates, termed in some contexts as Potential of Mean Force (PMF) (40). PMF captures thermodynamic changes undergone by the molecule during the unfolding. One of the most challenging feature of SMD is the gap between the experimental velocity values and those used computationally. Higher values of the velocity lead to an overestimation of the rupture forces (41). Nevertheless, SMD offers an atomistic picture of the structural changes during the unfolding and its results can be complemented with other calculations like the PMF. Mechanical unfolding of quadruplexes has been studied computationally with atomic detail in a few works (42,43). Li *et al*. (42) studied the unfolding of the parallel human telomeric DNA and estimate the unfolding free energy through Jarzynski equality (44). They showed that the unfolding pathway depends on the pulling-force application site. Likewise, Yang *et al*. (43) studied the unfolding of the thrombin binding aptamer (a structure formed by two G-tetrads that stack the divalent cation $\rm {Sr}^{2+}$) and showed that the largest contribution to the unfolding free energy comes from the removal of the central ion.

In this article, we provide the first comparative study of the mechanical unfolding of different short segments of human telomeric DNA and RNA G-quadruplex with different topologies, specifically the parallel, hybrid I and antiparallel configurations of the DNA and the parallel configuration of RNA in the presence of the central cations between each couple of quartets. In agreement with previous studies, the equilibrium simulations confirm that the central ions are important to maintain the folded structure of the G-quadruplexes, particularly for the parallel one. The influence of the central ions in the unfolding path and the rupture forces are then studied. We show that rupture events are strongly related to the coordination between the central ions and the guanines. Finally, the PMF is reconstructed to compare the main results obtained with the pulling simulations.

## MATERIALS AND METHODS

### MD simulation

**Figure 1.**
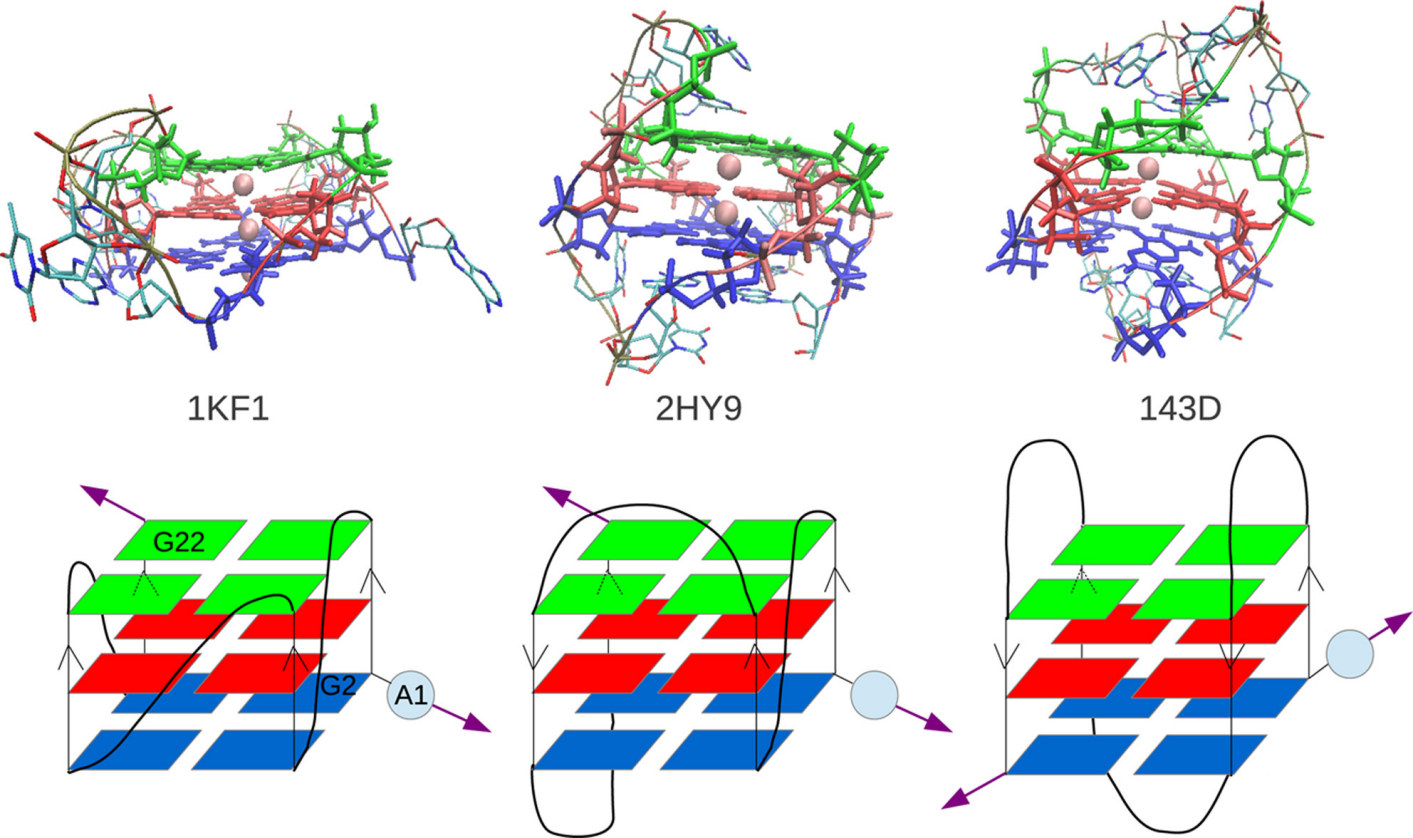
Simulated G4 structures. *Top:* All atom representation of the PDB files: 1KF1 ↑↑- parallel DNA, 2HY9 ↑↑↓ - hybrid I, 143D ↑↓ - antiparallel DNA. *Bottom:* Simplified representation of the structures. Guanines of the same tetrad are represented by parallelograms with the same colour. The position of the adenine A1 and guanines G2 and G22 bases are indicated. For sake of simplicity the bases of the loops are not represented. The violet arrows represent the direction of the pulling force. RNA is constructed from 1KF1 file as explained in the text.

The starting structures for the simulation have been downloaded from the Protein Data Bank. The items used are the following: the parallel intramolecular DNA with PDB id:1KF1 (12) (obtained by X-ray diffraction); the hybrid intramolecular DNA with PDB id:2HY9 (17) (obtained by NMR) and the antiparallel intramolecular DNA with PDB id:143D (13) (obtained by NMR). The first adenine base of 2HY9 was deleted. Thus all the structures have the same sequence composition: $\rm {d[AG_3(T_2AG_3)_3]}$. We manually build the atomic positions file for the parallel unimolecular RNA. For this, we make the following modifications to the parallel DNA 1KF1 file using the software VMD (45): an OH group is added to each $\rm {C2}^{\prime }$ sugar atom (to transform deoxyribose into ribose sugar) and replace the methyl group $\rm {CH_3}$ of each thymine by a single hydrogen atom (to transform thymine into uracil base). All structures have two $\rm {K^+}$ ions in the central channel along the axis of the quadruplex. We have used $\rm {K}^+$ ions instead of the $\rm {Na}^+$ in the antiparallel DNA in order to have similar conditions in all simulations. The structures of each G-quadruplex are shown in Figure 1. We use the following nomenclature for each G-quadruplex: 1KF1 ↑↑, for the parallel DNA; 2HY9 ↑↑↓, for the hybrid DNA; 143D ↑↓, for the antiparallel intramolecular DNA and RNA ↑↑, for the parallel RNA.

The MD simulations were carried out with the software GROMACS 4.6.5. (46) All the results present here were obtained with the modification Parmbsc0 of the Amber force field (47) although Amber 99 (48) was also probed. The structures were solvated with TIP3P water molecules in a periodic box of $6.5\,\rm {nm}\times 6.5\,\rm {nm} \times 24\,\rm {nm}$. Additional $\rm {K}^+$ ions were added to the system to counteract the negative phosphate charges of the DNA and RNA chains. The energy minimization involves 1000 steps of the steepest descent and 1000 steps of conjugated gradient algorithms. Then, the equilibration simulations have been conducted during 100 ps at 300 K in the NVT ensemble (constant number of particles, volume and temperature) followed by 100 ps at 300 K and 1 atm in the NPT ensemble (constant number of particles, pressure and temperature). In these equilibration simulations the G-quadruplex atoms were constrained in their initial positions, whereas the water molecules and the ions were allowed to move. The system temperature and pressure were kept fixed by using the modified Berendsen thermostat (51) and the Parrinello-Rahman barostat (52), respectively. Equilibrium MD simulations were carried out with Langevin dynamics for 45 ns with a damping factor of *m*/τ, where *m* is the mass of each atom involved in the dynamics and $\tau =0.1\,\rm {ps}$ the coupling constant time. The integration time step was 2 fs. Long-range electrostatic interactions were treated using the particle mesh Ewald (PME) approach with a 1 nm direct space cutoff. The Lennard-Jones interactions were cut off at a distance of 1 nm. To avoid any net movement of the DNA/RNA molecule in the box, the centre-of-mass displacement was removed at every time step. The default values of the Lennard-Jones interaction parameters for the $\rm {K}^+$ ions were used: $\epsilon =1.37 \times 10 ^ {-3} \,\rm {kcal/mol}$ and $\sigma =4.73$Å.

During the pulling simulations a backbone atom of one end is maintained fixed and a backbone atom at the other end is pulled with an harmonic spring. For this, we choose the $\rm {O3}^{\prime }$ and $\rm {O5}^{\prime }$ atoms of the G22 and A1 bases, respectively. The pulling parameters are taken from the work of Li *et al*. (42) which are typical values in such simulations: a force constant, $k_0=1000\,\rm {kJ/(mol \cdot nm^2)}$, and a pulling velocity, $v=1\,\rm {nm/ns}$. The pulling is performed along the direction $z$. In the initial steps of the simulation the molecule orients in such a way that the vector joining the fixed and the pulled atoms lies along the $z$ axis. The initial configurations for the pulling simulation are those obtained at the end of the equilibrium simulations. The trajectory was monitored during $8\,\rm {ns}$.

### PMF

The PMF maps the free energy of a given system along one or more reaction coordinates. Here, we chose as the reaction coordinate the $z$ distance between the same terminal atoms used to conduct the pulling simulations described above. The PMF was reconstructed then by using umbrella sampling (49) and the weighted histogram analysis method (50). The method consists in sampling the reaction coordinate by constraining the $z$ values around the minimum $z$_*i*_ of a biased harmonic potential. The sampling of $z$ is done inside *N* windows centred in $z$_*i*_ which are separated by a step distance *dz* = 0.2 nm. The biased potentials holding the chain are *W*($z$) = *k*_0_/2($z$ − $z$_*i*_)^2^ (*i* = 1, .., *N*), where the value of *k*_0_ is the same used in the pulling simulations. The $z$ distributions from the *N* windows obtained in thermalised conditions are then combined to get the PMF through the weighted histogram analysis method, by using a bin width of 0.08 nm. The errors in the estimation of the PMF were calculated by the Bayesian bootstrap analysis, both implemented in GROMACS 4.6.5 (46,53). In order to ensure good converged PMFs we used different thermalization and sampling times.

## RESULTS AND DISCUSSION

### Equilibrium simulations

Equilibrium simulations were conducted during 45 ns in order to check the stability of the G-quadruplexes and therefore the validity of the force field. For this, we calculated the Root Mean Square Deviation (RMSD) for both the stem and the loop atoms and the number of hydrogen bonds in each plane. The number of hydrogen bonds is defined based on cutoffs for the distance between the donor and the acceptor atoms (<0.35 nm) and for the angle formed by the hydrogen, donor and the acceptor atoms (<30 degrees) (46).

**Figure 2.**
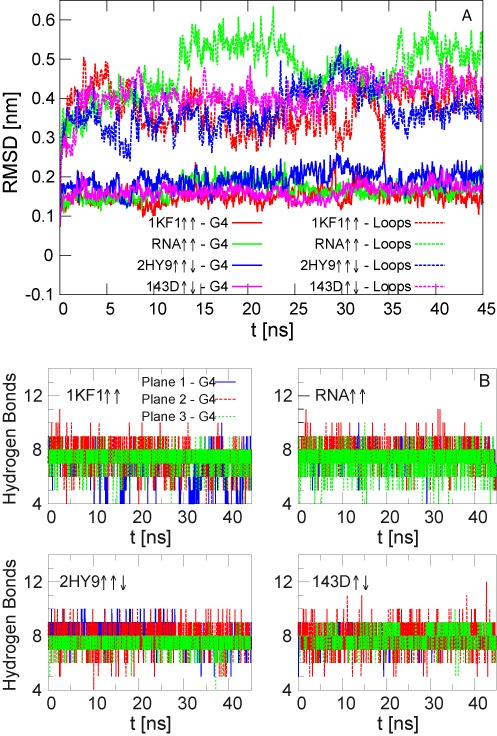
Equilibrium simulations with the central ions for the DNA and RNA quadruplexes. (**A**) RMSD of the stem (G4) and loop atoms. (**B**) Number of hydrogen bonds in each plane. The colour of the lines corresponds to the planes represented in Figure 1.

Figure 2 shows the RMSD values for the stem (G4) and the loop atoms for the different quadruplexes. It is observed that in all simulations the stem of both structures is quite stable while the loops deviate from the initial configuration. The position of the two central $\rm {K}^+$ ions is also stable over the simulation time and there is no exchange with the ions of the solution. The number of hydrogen bonds in each quartet shows fluctuations around the equilibrium value 8. Values higher than 8 indicate the formation of bifurcated hydrogen bonds in the plane, and lower ones correspond to temporal breaking of the bonds.

The model built for the unimolecular RNA was validated by comparing it with the dimeric experimental structure 3IBK obtained by X-ray diffraction (19). There is a good correspondence between the two models as shown by the superposition of their structures (Supplementary Figure S1) although the torsion angles of the backbone do not coincide for all the atoms. Equilibrium simulations of both unimolecular and dimeric RNA show formation of hydrogen bonds between the OH groups and the rest of the molecule.

The stability of the quadruplex structures was further investigated through the Root Mean Square Fluctuations (RMSF) that quantifies the average deviation of each atom during a given trajectory. This magnitude corroborates also the higher flexibility of the loops as compared with the quadruplex stem (Supplementary Figure S2). Overall, the equilibrium simulations show that all the G-quadruplexes are stable at the beginning of the pulling simulation.

**Figure 3.**
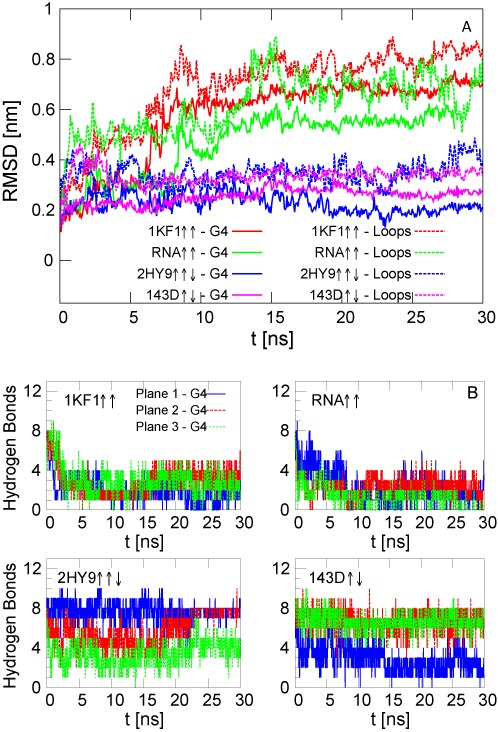
Equilibrium simulations for the DNA intramolecular G-quadruplexes without the ions in the central channel. (**A**) RMSD of the stem (G4) and loop atoms. (**B**) Number of hydrogen bonds in each quartet

We have also analysed the behaviour of the G- quadruplexes without the central ions. It is well known that the central ions enhance the stability of the quadruplex structure (36–39). In agreement with these studies, we have obtained that all structures become unstable in comparison to those with the central ions, as visible from both the higher values of the RMSD and the decrease of hydrogen-bond number as a function of the time (see Figure 3). The way each G-quadruplex is distorted in the absence of the central ions is also different. The highest deviations with respect to the initial configuration are obtained for the two parallel structures 1KF1 ↑↑ and RNA ↑↑ (see also Supplementary Figure S3 where one graphical example of the conformations at the end of the equilibrium simulations has been presented). This effect was previously studied by MD in (39). In that work, it was shown that strand slippage is easier to occur in the parallel structure with all guanines in the *anti* conformation, whereas in the antiparallel structure the alternate conformations of guanines *syn/anti* prevent strand slippage conferring a higher stiffness to the quartet stem. In some of the realizations without the central cations, one of the ions of the solution enters inside the quadruplex channel leading to the reorganization of the structure. One example is for the hybrid quadruplex 2HY9, where one ion is captured around $t=23 \,\rm {ns}$ (Figure 3B) and the number of the hydrogen bonds of each plane returns to its equilibrium value of 8. Similar to the simulations with the central ions, the OH group of the ribose sugar of the RNA forms several intramolecular hydrogen bonds. However, these interactions are not able to stabilise the quadruplex conformation.

### Pulling simulations

**Figure 4.**
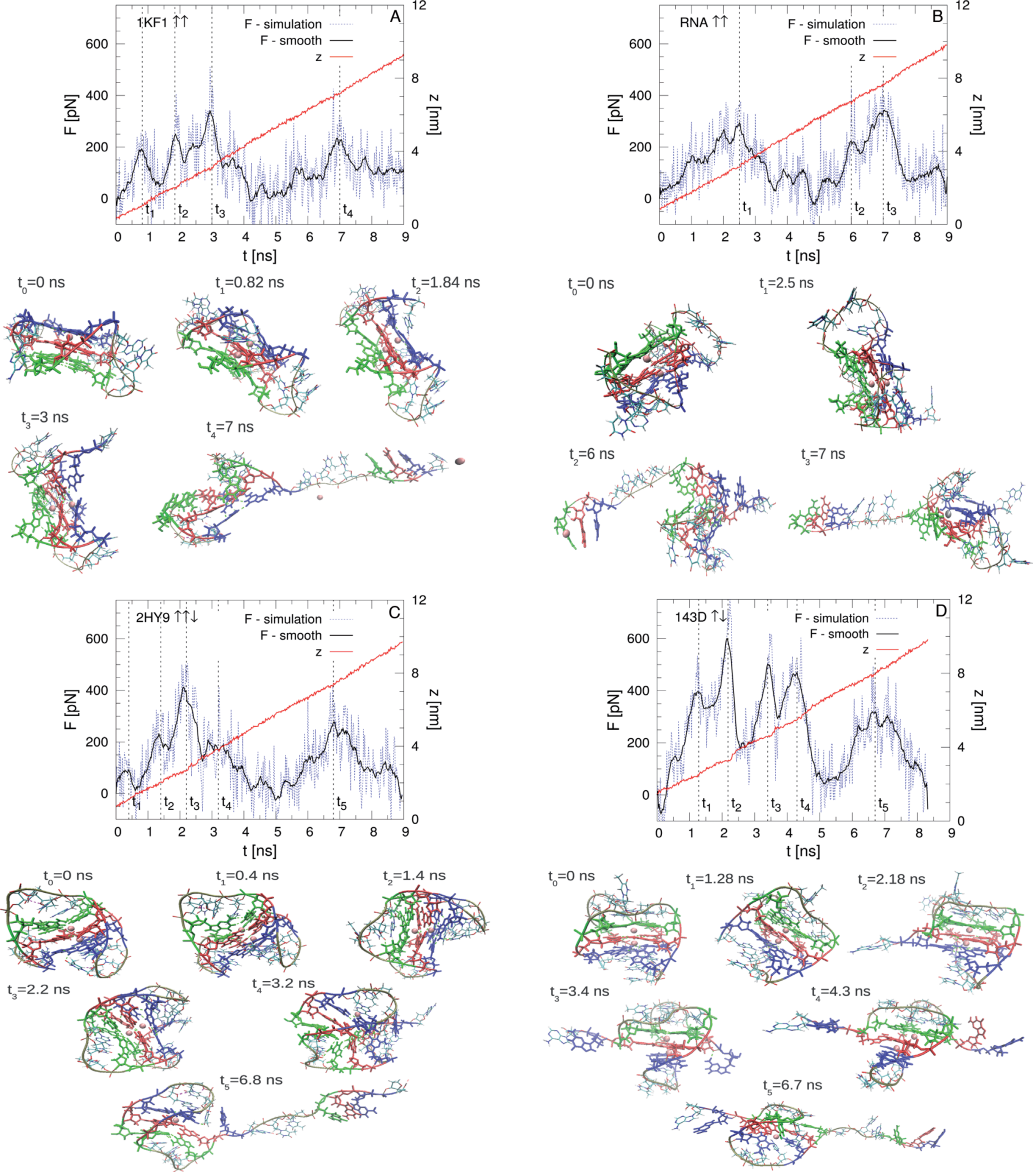
Force and extension as a function of the time during the pulling simulations. Vertical dashed lines indicate the time at which the snapshots are presented. (**A**) Parallel 1KF1 ↑↑. (**B**) Parallel RNA ↑↑. (**C**) Hybrid 2HY9 ↑↑↓. (**D**) Antiparallel 143D ↑↓. In the snapshot *t*_3_ of RNA the represented ion is coloured in silver to indicate that is from the solution.

SMD simulations are started from the final structures obtained in the equilibrium simulations. The force at the pulling spring and the $z$ distance between the pulled and the fixed atoms are computed as a function of the simulation time (Figure 4). The curves display a fine structure of rupture events characterised by high-amplitude force oscillations as a function of the time. Smoothed force-extension curves are also plotted in the same graphs for the sake of comparison. Some snapshots at the times indicated by the dashed lines are also presented.

There are differences concerning the force values and the rupture patterns among the different quadruplexes. Antiparallel quadruplex exhibits the highest rupture forces similar to the experimental results for quadruplexes with four guanine planes (22,23). Also, the unfolding path of the antiparallel quadruplex is clearly the most stepwise as visible from the presence of sharp force peaks which reveal an unfolding dynamics made by subsequent disruptions.

**Figure 5.**
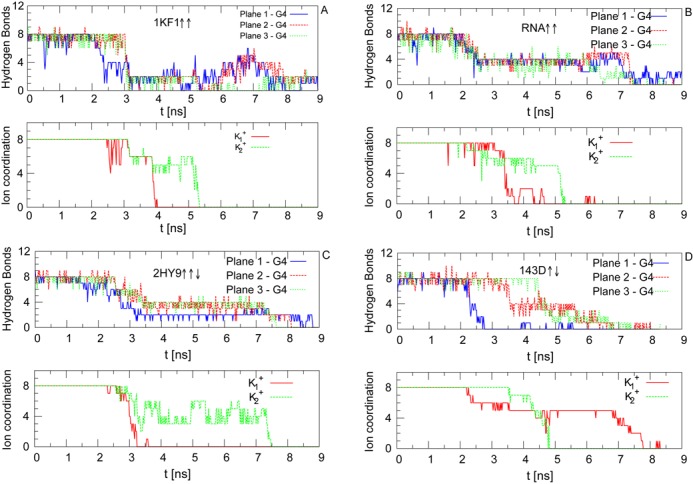
Number of hydrogen bonds in each plane (top panels) and coordination number of the central ions (bottom panels) for the structures: (**A**) Parallel 1KF1 ↑↑; (**B**) Parallel RNA ↑↑; (**C**) Hybrid 2HY9 ↑↑↓; (**D**) Antiparallel 143D ↑↓.

To have a better description of the unfolding pathway we calculated the number of the hydrogen bonds in each quartet and the coordination number of each central ion with its neighbor guanines. The coordination number was evaluated through the distances between each central ion and the O6 atoms of each of its eight neighboring guanines (these distances are shown in Supplementary Figure S4). In the folded structure, where the coordination is maximum and equals 8, these distance values fluctuate around 0.35 nm with amplitudes below 0.1 nm. Then, we can say that a guanine is not coordinated to the central ion if the distance between them is larger than 0.45 nm. Figure 5 shows that in the parallel and hybrid structures the three guanine quartets break almost simultaneously, as can be seen from the decrease of the number of hydrogen bonds in each plane, while in the antiparallel one, the rupture of the planes occurs at different stages of the simulation. When the quadruplex unfolds, the ion-guanine distance increases and the coordination number decreases. For the parallel and the hybrid quadruplex, the coordination numbers of $\rm {K_1^+}$ and $\rm {K_2^+}$ decrease almost simultaneously at *t* ≈ 3 ns. In the antiparallel case, the decrease of the coordination number presents instead longer steps.

It is worth noting that the structural changes in the unfolding trajectories observed in Figure 5 are in close correspondence with the force curves displayed in Figure 4. In fact, the instants at which the rupture events are observed ($t_3=3 \,\rm {ns}$, $t_1=2.5 \,\rm {ns}$ and $t_3=2.2 \,\rm {ns}$ for parallel DNA, parallel RNA and hybrid DNA respectively, and $t_2=2.18 \,\rm {ns}$, $t_3=3.4 \,\rm {ns}$ and $t_4=4.3 \,\rm {ns}$ for antiparallel DNA) match those of the decrease of the number of hydrogen bonds (top panels of Figures 5A–D) and the loss of coordination of the central ions (bottom panels of Figures 5A–D). The highest force peaks are obtained in all cases when the ions are in the central channel of the quadruplexes (see the snapshots of Figure 4). These forces come, of course, not only from the disruptions of the ion-guanine interactions but also from the simultaneous breaking of the hydrogen bonds and stacking interactions. What is important is that the ions coordinate a favorable arrangement of the quadruplex structure and then are able to increase the strength of all the interactions. The influence of different non covalent bonds in quadruplex have been studied in detail with quantum calculations in (55). It was shown there that the ion-guanine interactions increase the cooperativity and the strength of hydrogen bonds and stacking forces. A similar effect is derived from our MD simulations even if only the Coulomb and Lennard-Jones potentials are used to model the interactions between the ions and the quadruplex.

The subsequent ruptures observed in the unfolding of the antiparallel quadruplex are also related to the geometry itself, i.e. which planes are under tension during the pulling. In fact, in the antiparallel quadruplex the Plane 1 (the blue one in Figure 1) is directly under tension from two of its edges and then is disrupted first as shown in Figure 5D where the hydrogen bonds break before than in the other planes. Differently, for the parallel and hybrid quadruplexes the tension is applied between the Plane 1 and Plane 3 (the blue and green ones) and then break almost at the same time and with slower progression in time, see Figure 5A–C. Under those pulling conditions the central ions can leave the channel easier and then the rupture occurs at lower forces and in a more cooperative way.

The rupture forces found for the parallel DNA and the constructed RNA quadruplexes are very similar. This result does not agree with experimental works where higher mechanical stability has been obtained for RNA quadruplex (25). Our results could be related to the molecular model we have used for RNA where the backbone torsion angles are not exactly the same as in the experimental dimeric structure. In the calculations of the hand made monomeric RNA we have checked the temporal evolution of the hydrogen bonds formed by the $\rm {OH}$ groups. These interactions are proposed to be the responsible for the higher mechanical stability of RNA with respect to DNA (19). However, from our simulations there is not a clear correlation between the force curve in the unfolding process and the number of these hydrogen bonds. In fact, as visible in Supplementary Figure S5 the number of hydrogen bonds along the unfolding trajectory remains close to that obtained from the equilibrium simulations. These interactions can confer conformational stability to the quadruplex but seem to have small influence in the values of the rupture force.

In all the trajectories of the four studied quadruplexes, we observe that one of the unfolded steps is a triplex like structure in which one of the four strands of the quadruplex stem and one loop is unfolded while the rest of the structure is folded (see the snapshots of Figures 4A–D at times $t_4=7 \,\rm {ns}$, $t_3=7 \,\rm {ns}$, $t_5=6.8 \,\rm {ns}$ and $t_5=6.7 \,\rm {ns}$, respectively). The rupture of this last structure is responsible for the force peaks observed in Figure 4 at those times. This triplex structure has been proposed as an intermediated state during the mechanical unfolding of telomeric DNA and RNA quadruplexes in (25,54). Our simulations show that this triplex state can have different arrangements depending on the quadruplex geometry and the presence or not of the ions.

The role of the central ions in the unfolding path is also corroborated by the pulling simulations without these ions (Supplementary Figure S6). The starting structures for these calculations are those obtained at the end of the equilibrium simulations described in the previous section. In these cases, the removal of the ions causes that the three consecutive guanines of one side of the stem can be pulled out the quadruplex structure almost at the same time, as demonstrated by the decrease of the hydrogen number in the three planes (see Supplementary Figure S6B). This behaviour suggests that the stacking between consecutive planes is significantly reduced in the absence of the ions and for this reason, as visible in Supplementary Figure S6A, there is a reduction on the values of rupture forces and a change on the unfolding pattern. Besides, the removal of the central ions eliminates the geometrical constraints and allows the formation of structures where guanines are no longer disposed on the same plane. The disruptions of such structures give rise to the peaks observed for the antiparallel and hybrid conformations in Supplementary Figure S6A.

To check the reproducibility of our results, we performed some extra realizations of each pulling experiment with different seeds in the integration of the Langevin equations (see Supplementary Figure S7 where the different curves of the force as a function of the time are reported). The main qualitative behaviours are maintained: the rupture forces are always higher for the antiparallel conformation and the force pattern is more stepwise than for the other configurations. Taken the maximum rupture force as a measure of the mechanical stability we get that the most stable quadruplex is the antiparallel, followed by the hybrid and the parallel (DNA and RNA) quadruplexes. This order is in agreement with the mechanical stabilities obtained from the unfolding free energies of telomeric DNA quadruplexes reported in (24).

As explained in the methods section, the antiparallel structure has been experimentally observed in solution with $\rm {Na}^+$ instead of $\rm {K}^+$. For this reason, we have also simulated the unfolding of the antiparallel structure with two $\rm {Na}^+$ ions in the channel of the quadruplex. Supplementary Figure S7 shows that the stepwise pattern also holds in this case. The rupture forces decrease with respect to those with $\rm {K}^+$ ions but are still higher than in the other quadruplexes.

In general, the rupture forces obtained in these simulations are higher than the experimental ones. This is due to the much higher velocity that is required in the numerical simulations. Lower velocity values allow thermal fluctuations to aid the mechanical unfolding and then lower forces are obtained (41). However, as we will discuss later, the qualitative picture of the unfolding obtained here can be validated with calculations that do not depend on the pulling velocity. Another aspect, related to the temporal scales of the simulations, is that the pulling is performed in few nanoseconds, well below the microsecond scale time in which the main conformational changes of the loops are observed (33). In order to explore different geometries of the loops in relatively short times, we have performed equilibrium calculations at higher temperatures (400 K). In the simulation times at this temperature the mobility of the loop increases but the quadruplexes remain folded. We have selected some of the structures along these trajectories as starting conformations for the pulling simulations at room temperature (300 K). Also in this case, the values of the force and the unfolding patterns obtained do not change significantly.

### Reconstruction of the PMF of DNA quadruplexes

**Figure 6.**
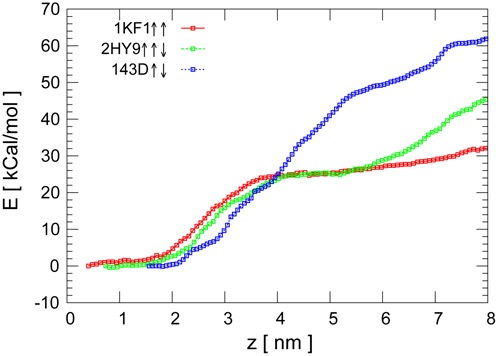
PMF for the DNA quadruplexes.

The thermodynamic changes undergone by the G-quadruplex during the pulling can be captured in the PMF, which evaluates the free energy along the reaction coordinate, *E*($z$). The PMF is reconstructed by using umbrella sampling and the weighted histogram analysis method. The PMF provides another approach to compare the relative mechanical stability between the different quadruplexes. In order to have reliable estimates of the free energy differences the system has to achieve a state of thermal equilibrium and the sampling has to be big enough to capture all the relevant molecular structures consistent with $z$_*i*_. For this reason, we have used different thermalization and sampling times to ensure the convergence of the PMF curves. We have selected profiles with 4 ns and 40 ns of thermalization and sampling times, respectively. The converged profiles are shown in Figure 6 for the DNA G-quadruplexes. As parallel DNA and RNA have similar rupture patterns we have not computed the PMF for the RNA quadruplex. The errors in the reconstruction of the PMF were estimated by using Bayesian bootstrap analysis (Supplementary Figure S8).

The structure of the PMF consists in successive concave and convex regions. Convexity demarcates mechanically stable conformations while concavity points to unfolding events. The antiparallel G-quadruplex exhibits a profile with alternate convex and concave regions corresponding to intermediate structural states and unfolding events during the stretching, respectively. These steps are in correspondence with the sharp peaks observed in the force curves. A similar profile was obtained with this computational method for the thrombin binding aptamer (43) showing that the antiparallel geometry tends to unfold in a stepwise fashion. In the parallel and hybrid configurations only one unfolding event is clearly distinguished. Some of the representative structures obtained along the reaction coordinate $z$ for the antiparallel quadruplex are shown in Supplementary Figure S9. These snapshots were selected from the equilibrium simulations performed in different windows during the umbrella sampling. It is evident from these snapshots that the central ions coordinate different intermediated states of the antiparallel structure.

The qualitative unfolding behaviour obtained by the PMF is therefore in agreement with that derived from the pulling simulations, which were obtained at pulling velocities higher than the experimental ones. To be more specific, from the PMF profiles we are able to estimate the quasi-static force vs. extension curves, and to include in them the influence of different values of the elastic constant *k*_0_ as shown in Supplementary Figure S10. The numerical results of this figure are in qualitative correspondence with the force curves obtained in the pulling simulations in Figure 4.

The experimental values of the unfolding free energy for the human telomeric quadruplex reported in ref (24), obtained by using Jarzynski equality (44), are lower than those obtained in our simulations with the umbrella sampling method (experimental values are in the order of 6–11 kcal/mol). These differences may be due to very long equilibrium calculations required in each of the umbrella sampling windows. Moreover, the energy profile for the antiparallel structure with $\rm {Na}^+$ should be lower than the current with $\rm {K}^+$ as expected from the force curves obtained in the previous section. Even in the presence of these discrepancies the calculated energy profiles give us another comparative measure about the relative mechanical stability among the different quadruplexes. From the inspection of Figure 6 it is confirmed that the mechanical stability of the antiparallel structure is higher than the hybrid, and the latter higher than the parallel one. All these results are in agreement with the pulling simulations above reported as well as with the experimental results. In addition, the stepwise pattern of the antiparallel PMF may be also consistent with the possible existence of free energy barriers during the mechanical unfolding of the different planes of the G-quadruplex, as suggested by de Messieres *et al*. (23) and Ghimire *et al*. (56).

## CONCLUSIONS

We have made a comparative and systematic study of the mechanical unfolding of different conformations of the telomeric DNA and RNA quadruplexes by using all atom MD simulations. The role of the central ions in both the stability and the mechanical unfolding of these quadruplexes has been addressed. All the configurations exhibit similar stability when the $\rm {K}^+$ ions are within the central G-quadruplex channel and experience a significant weakening when they are removed.

Further, SMD simulations reveal that the unfolding path of a G-quadruplex strongly depends on its topology. The rupture forces registered during the unfolding for the antiparallel DNA structure are higher than those of the hybrid and the parallel ones, in agreement with experimental results. Moreover, the rupture force-time profile of the antiparallel G-quadruplex displays a stepwise pattern which is not apparent for the other structures. These features are related to the higher stability due to the presence of the $\rm {K}^+$ ions which remain longer in the central positions of the G-quadruplex channel. DNA and RNA parallel quadruplexes exhibit similar mechanical stabilities.

The pulling velocities used in the simulations are several orders of magnitude higher than those used experimentally. For this reason, the rupture forces obtained here are overestimated. However, the qualitative rupture profiles obtained from the pulling simulation are in agreement with those derived from the PMF, indicating that the simulations provide a representative information about the unfolding pathways of G-quadruplex structures.

## SUPPLEMENTARY DATA

Supplementary Data are available at NAR Online.

SUPPLEMENTARY DATA

## References

[1] Burge S., Parkinson G.N., Hazel P., Todd A.K., Neidle S. (2006). Quadruplex DNA: sequence, topology and structure. Nucleic Acids Res..

[2] Collie G.W., Parkinson G.N. (2011). The application of DNA and RNA G-quadruplexes to therapeutic medicines. Chem. Soc. Rev..

[3] Lim K.W., Ng V.C., Martin-Pintado N., Heddi B., Phan A.T. (2013). Structure of the human telomere in Na+ solution: an antiparallel (2+ 2) G-quadruplex scaffold reveals additional diversity. Nucleic Acids Res..

[4] Lam E.Y., Beraldi D., Tannahill D., Balasubramanian S. (2013). G-quadruplex structures are stable and detectable in human genomic DNA. Nat. Commun..

[5] Siddiqui-Jain A., Grand C.L., Bearss D.J., Hurley L.H. (2002). Direct evidence for a G-quadruplex in a promoter region and its targeting with a small molecule to repress c-MYC transcription. Proc. Natl. Acad. Sci. U.S.A..

[6] Azzalin C.M., Reichenbach P., Khoriauli L., Giulotto E., Lingner J. (2007). Telomeric repeat-containing RNA and RNA surveillance factors at mammalian chromosome ends. Science.

[7] Wieland M., Hartig J.S. (2007). RNA quadruplex-based modulation of gene expression. Chem. Biol..

[8] Schoeftner S., Blasco M.A. (2008). Developmentally regulated transcription of mammalian telomeres by DNA-dependent RNA polymerase II. Nat. Cell. Biol..

[9] Sun D., Thompson B., Cathers B.E., Salazar M., Kerwin S.M., Trent J.O., Jenkins T.C., Neidle S., Hurley L.H. (1997). Inhibition of human telomerase by a G-quadruplex-interactive compound. J. Med. Chem..

[10] Mergny J.-L., Hélène C. (1997). G-quadruplex DNA: A target for drug design. Nat. Med..

[11] Horard B., Gilson E. (2008). Telomeric RNA enters the game. Nat. Cell. Biol..

[12] Parkinson G.N., Lee M.P., Neidle S. (2002). Crystal structure of parallel quadruplexes from human telomeric DNA. Nature.

[13] Wang Y., Patel D.J. (1993). Solution structure of the human telomeric repeat d [AG_3_(T_2_ AG_3_)_3_] G-tetraplex. Structure.

[14] Phan A.T., Kuryavyi V., Luu K.N., Patel D.J. (2007). Structure of two intramolecular G-quadruplexes formed by natural human telomere sequences in K+ solution. Nucleic Acids Res..

[15] Dai J., Carver M., Punchihewa C., Jones R.A., Yang D. (2007). Structure of the Hybrid-2 type intramolecular human telomeric G-quadruplex in K+ solution: insights into structure polymorphism of the human telomeric sequence. Nucleic Acids Res..

[16] Dai J., Carver M., Yang D. (2008). Polymorphism of human telomeric quadruplex structures. Biochimie.

[17] Dai J., Punchihewa Ch., Ambrus A., Chen D., Jones R.A., Yang D. (2007). Structure of the intramolecular human telomeric G-quadruplex in potassium solution: a novel adenine triple formation. Nuc. Acid. Res..

[18] Martadinata H., Phan A.T. (2009). Structure of propeller-type parallel-stranded RNA G-quadruplexes, formed by human telomeric RNA sequences in K+ solution. J. Am. Chem. Soc..

[19] Collie G.W., Haider S.M., Neidle S.Y., Parkinson G.N. (2010). A crystallographic and modelling study of a human telomeric RNA (TERRA) quadruplex. Nucleic Acids Res..

[20] Garavís M., Bocanegra R., Herrero-Galán E., González C., Villasante A., Arias-Gonzalez J.R. (2013). Mechanical unfolding of long human telomeric RNA (TERRA). Chem. Commun..

[21] Koirala D., Dhakal S., Ashbridge B., Sannohe Y., Rodriguez R., Sugiyama H., Balasubramanian S., Mao H. (2011). A single-molecule platform for investigation of interactions between G-quadruplexes and small-molecule ligands. Nat. Chem..

[22] Yu Z., Schonhoft J.D., Dhakal S., Bajracharya R., Hegde R., Basu S., Mao H. (2009). ILPR G-quadruplexes formed in seconds demonstrate high mechanical stabilities. J. Am. Chem. Soc..

[23] Messieres M., Chang J.Ch., Brawn-Cinani B., La Porta A. (2012). Single-molecule study of G-quadruplex disruption using dynamic force spectroscopy. Phys. Rev. Lett..

[24] Dhakal S., Cui Y., Koirala D., Ghimire Ch., Kushwaha S., Yu Z., Yangyuoru P.M., Mao H. (2013). Structural and mechanical properties of individual human telomeric G-quadruplexes in molecularly crowded solutions. Nucl. Acids Res..

[25] Yangyuoru P.M., Zhang A.Y.Q., Shi Z., Koirala D., Balasubramanian S., Mao H. (2013). Mechanochemical properties of individual human telomeric RNA (TERRA) G-quadruplexes. Chem. Bio. Chem..

[26] Arias-Gonzalez J.R. (2014). Single-molecule portrait of DNA and RNA double helices. Integr. Biol..

[27] Lane A.N. (2012). The stability of intramolecular DNA G-quadruplexes compared with other macromolecules. Biochimie.

[28] Gray R.D., Trent J.O., Chaires J.B. (2014). Folding and unfolding pathways of the human telomeric G-quadruplex. J. Mol. Biol..

[29] Mashimo T., Yagi H., Sannohe Y., Rajendran A., Sugiyama H. (2010). Folding pathways of human telomeric type-1 and type-2 G-quadruplex structures. J. Am. Chem. Soc..

[30] Bian Y., Tan C., Wang J., Sheng Y., Zhang J. (2014). Atomistic picture for the folding pathway of a hybrid-1 type human telomeric DNA G-quadruplex. PLoS Comput. Biol..

[31] Spackova N., Berger I., Sponer J. (1999). Nanosecond molecular dynamics simulations of parallel and antiparallel guanine quadruplex DNA molecules. J. Am. Chem. Soc..

[32] Li M.H., Luo Q., Xue X.G., Li Z.S. (2010). Toward a full structural characterization of G-quadruplex DNA in aqueous solution: Molecular dynamics simulations of four G-quadruplex molecules. J. Mol. Struct-Theochem..

[33] Islam B., Sgobba M., Laughton Ch., Orozco M., Sponer J., Neidle S., Haider S. (2013). Conformational dynamics of the human propeller telomeric DNA quadruplex on a microsecond time scale. Nucleic Acids Res..

[34] Heddi B., Phan A.T. (2011). Structure of human telomeric DNA in crowded solution. J. Am. Chem. Soc..

[35] Martadinata H., Heddi B., Lim K.W., Phan A.T. (2011). Structure of long human telomeric RNA (TERRA): G-quadruplexes formed by four and eight UUAGGG repeats are stable building blocks. Biochemistry.

[36] Chowdhury S., Bansal M. (2001). G-quadruplex structure can be stable with only some coordination sites being occupied by cations: a six-nanosecond molecular dynamics study. J. Phys. Chem. B.

[37] Spackova N., Berger I., Sponer J. (2001). Structural dynamics and cation interactions of DNA quadruplex molecules containing mixed guanine/cytosine quartets revealed by large-scale MD simulations. J. Am. Chem. Soc..

[38] Cavallari M., Calzolari A., Garbesi A., Di Felice R. (2006). Stability and migration of metal ions in G4-wires by molecular dynamics simulations. J. Phys. Chem. B.

[39] Stadlbauer P., Krepl M., Cheatham T.E., Koca J., Sponer J. (2013). Structural dynamics of possible late-stage intermediates in folding of quadruplex DNA studied by molecular simulations. Nucleic Acids Res..

[40] Kirkwood J. (1935). Statistical mechanics of fluid mixtures. J. Chem. Phys..

[41] Hsin J., Strumpfer J., Lee E.H., Schulten K. (2011). Molecular origin of the hierarchical elasticity of titin: simulation, experiment, and theory. Annu. Rev. Biophys..

[42] Li H., Cao E., Gisler T. (2009). Force-induced unfolding of human telomeric G-quadruplex: a steered molecular dynamics simulation study. Biochem. Bioph. Res. Co..

[43] Yang C., Jang S., Pak Y. (2011). Multiple stepwise pattern for potential of mean force in unfolding the thrombin binding aptamer in complex with Sr2+. J. Chem. Phys..

[44] Jarzynski Ch. (1997). Nonequilibrium equality for free energy differences. Phys. Rev. Lett..

[45] Humphrey W., Dalke A., Schulten K. (1996). VMD: visual molecular dynamics. J. Molec. Graphics.

[46] Hess B., Kutzner C., van der Spoel D., Lindahl E. (2008). GROMACS 4: algorithms for highly efficient, load-balanced, and scalable molecular simulation. J. Chem. Theory Comp..

[47] Prez A., Marchn I., Svozil D., Sponer J., Cheatham T.E., Laughton Ch.A., Orozco M. (2007). How well does a restrained electrostatic potential (RESP) model perform in calculating conformational energies of organic and biological molecules. J. Comp. Chem.

[48] Wang J., Cieplak P., Kollman P. A. (2000). Refinement of the AMBER force field for nucleic acids: improving the description of α/γ conformers. Biophys. J..

[49] Torrie G.M., Valleau J.P. (1977). Nonphysical sampling distributions in Monte Carlo free-energy estimation: umbrella sampling. J. Comput. Phys..

[50] Kumar S., Bouzida D., Swendsen R.H., Kollman P.A., Rosenberg J.M. (1992). The weighted histogram analysis method for free-energy calculations on biomolecules. I. The method. J. Comput. Chem..

[51] Bussi G., Donadio D., Parrinello M. (2007). Canonical sampling through velocity rescaling. J. Chem. Phys..

[52] Parrinello M., Rahman A. (1981). Polymorphic transitions in single crystals: a new molecular dynamics method. J. Appl. Phys..

[53] Hub J.S., de Groot B.L., van der Spoel D. (2010). g_wham_—a free weighted histogram analysis implementation including robust error and autocorrelation estimates. J. Chem. Theory Comput..

[54] Li W., Hou X.M., Wang P.Y., Xi X.G., Li M. (2013). Direct measurement of sequential folding pathway and energy landscape of human telomeric G-quadruplex structures. J. Am. Chem. Soc..

[55] Yurenko Y.P., Novotn J., Sklen V., Marek R. (2014). Exploring non-covalent interactions in guanine-and xanthine-based model DNA quadruplex structures: a comprehensive quantum chemical approach. Phys. Chem. Chem. Phys..

[56] Ghimire C., Park S., Iida K., Yangyuoru P., Otomo H., Yu Z., Nagasawa K., Sugiyama H., Mao H. (2013). Direct quantification of loop interaction and π−π stacking for G-quadruplex stability at the submolecular level. J. Am. Chem. Soc..

