# Supplementary material for: Role of the central cations in the mechanical unfolding of DNA and RNA G-quadruplexes
Source: Nucleic Acids Res. 2015 Jul 13;43(15):7638–47. doi: 10.1093/nar/gkv690 (PMC4551928; doi:10.1093/nar/gkv690)
Supplement: SUPPLEMENTARY DATA [file supp_43_15_7638__index.html]

Role of the central cations in the mechanical unfolding of DNA and RNA G-quadruplexes — Role of the central cations in the mechanical unfolding of DNA and RNA G-quadruplexes — SUPPLEMENTARY DATA 

# Role of the central cations in the mechanical unfolding of DNA and RNA G-quadruplexes

## SUPPLEMENTARY DATA

- SUPPLEMENTARY DATA
