# Supplementary material for: Role of the central cations in the mechanical unfolding of DNA and RNA G-quadruplexes
Source: Nucleic Acids Res. 2015 Jul 13;43(15):7638–47. doi: 10.1093/nar/gkv690 (PMC4551928; doi:10.1093/nar/gkv690)
Supplement: SUPPLEMENTARY DATA [file supp_gkv690_nar-00469-f-2015-File002.pdf]

Role of the central cations in the mechanical unfolding of DNA and RNA G-quadruplexes

Ana Elisa Bergues-Pupo,[1,2,3] J. Ricardo Arias-Gonzalez,[4,5] María Carmen Morón,[1,6] Alessandro Fiasconaro,[7,*] and F. Falo[1,2]

[1]*Departamento de Física de la Materia Condensada, Facultad de Ciencias, Universidad de Zaragoza, Pedro Cerbuna 12, 50009 Zaragoza, Spain*

[2]*Instituto de Biocomputación y Física de Sistemas Complejos (BIFI), Universidad de Zaragoza, Mariano Esquillor, 50018 Zaragoza, Spain*

[3]*Departamento de Física, Universidad de Oriente, 90500 Santiago de Cuba, Cuba*

[4]*Instituto Madrileño de Estudios Avanzados en Nanociencia (IMDEA Nanociencia), Cantoblanco, 28049 Madrid, Spain*

[5]*CNB-CSIC-IMDEA Nanociencia Associated Unit "Unidad de Nanobiotecnología"*

[6]*Instituto de Ciencia de Materiales de Aragón (ICMA), Consejo Superior de Investigaciones Científicas, Universidad de Zaragoza, Pedro Cerbuna 12, 50009 Zaragoza, Spain*

[7]*School of Mathematical Sciences, Queen Mary University of London - Mile End Road, London E1 4NS, UK*

[*]Electronic address: `A.Fiasconaro@qmul.ac.uk`

Calculation of the average force from the Potential of Mean Force

The average equilibrium force needed to keep the end to end distance z of the molecule around the fixed extension z_i with an harmonic potential $V_{z_i}(z) = 0.5k_0(z - z_i)^2$ can be expressed as [1]:

$$\langle F \rangle_{z_i} = \frac{\int dz \frac{\partial V_{z_i}(z)}{\partial z} \exp\{-\beta[E(z) + V_{z_i}(z)]\}}{\int dz \exp\{-\beta[E(z) + V_{z_i}(z)]\}}, \tag{1}$$

where $E(z)$ is the PMF, $\beta = 1/k_B T$ and k_0 the elastic constant. The values of the forces derived from the PMF are lower than those from the pulling simulations (see Figure S10), although they are still larger than the experimental ones. As stated in the main text, we find good correspondence between the force patterns obtained by the pulling simulations and the PMF. It is interesting to note that the basic phenomenology observed during the pulling is dependent on the spring stiffness, which determines the precision in the detection of the stepwise ruptures and in the force measurements.

---

[1] I. Franco, M.A. Ratner, and G.C. Schatz, Nano and Cell Machines: Funadamental and Frontiers, (Wiley, Microsystem and Nanotechnology Series, 2002).

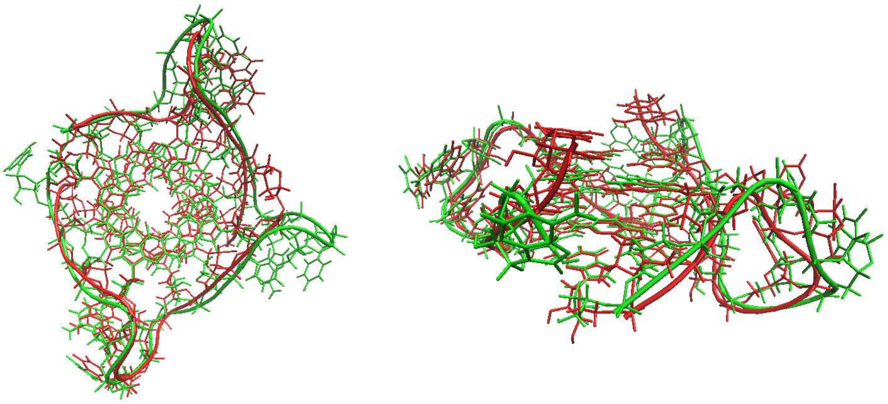

FIG. S1: Superposition of the experimental dimeric (red) and the hand made unimolecular (green) RNA models. *Left*: Top view. *Right*: Lateral view.

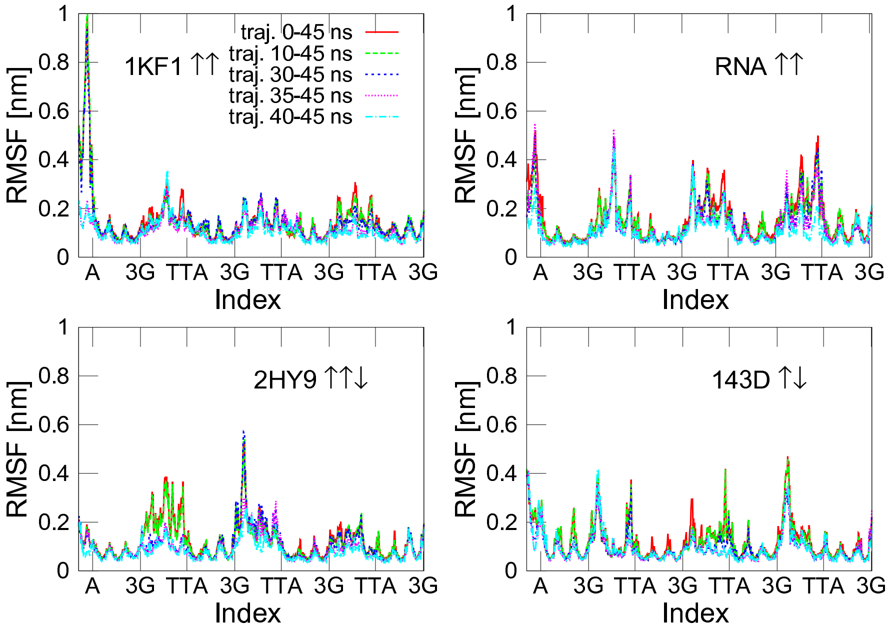

FIG. S2: **Equlibrium Simulations**. Root Mean Square Fluctuations (RMSF) in the equilibrium simulations with the central ions. RMSF is calculated for different lengths of the trajectory. The horizontal tics of the figure indicate the fragment of the chain that ends at this position. The largest fluctuations are localized at loop regions indicated by TTA tics.

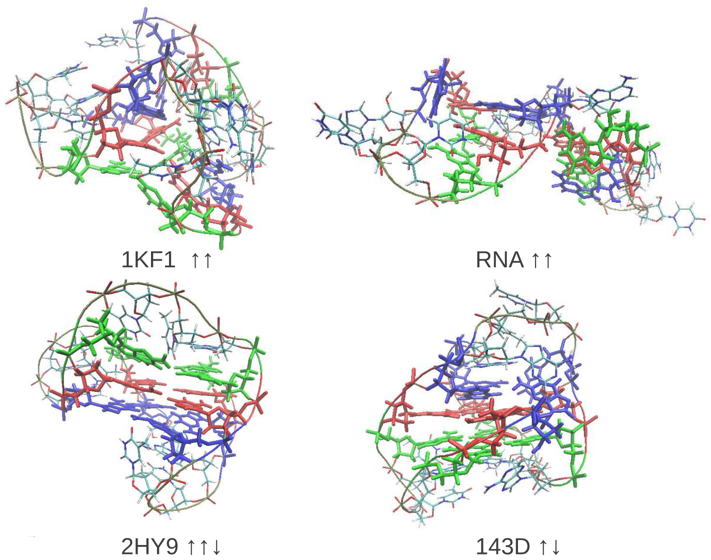

FIG. S3: **Equlibrium Simulations**. Structures at the end of the equilibrium simulations without the central ions. Guanines belonging to the same quartet are plotted in the same color.

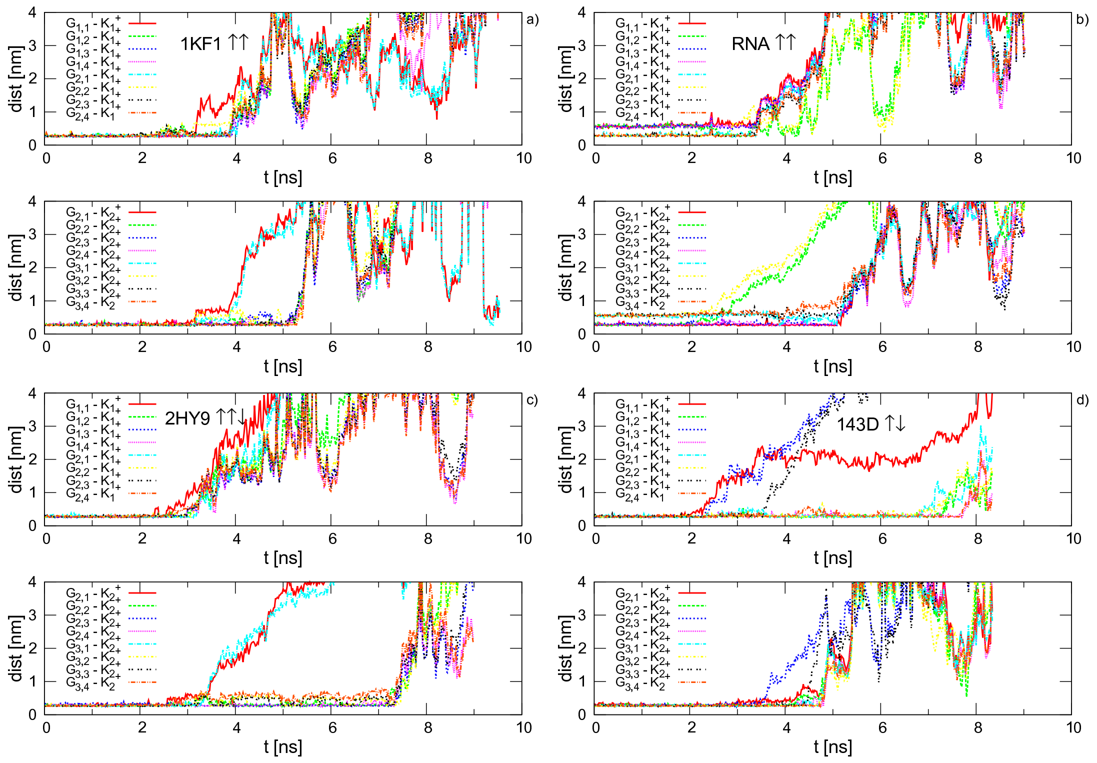

FIG. S4: **Pulling Simulations**. Distance between guanines and central ions used to define the coordination number of the central ion during the pulling simulations: a) 1KF1 $\uparrow\uparrow$ - Parallel. b) RNA $\uparrow\uparrow$ - Parallel. c) 2HY9 $\uparrow\uparrow\downarrow$ - Hybrid. d) 143D $\uparrow\downarrow$ - Antiparallel. The notation $\mathrm{G_{i,j}} - \mathrm{K}$ refers to the distance between the central ion and the O6 atom belonging to $j - th$ guanine of plane i.

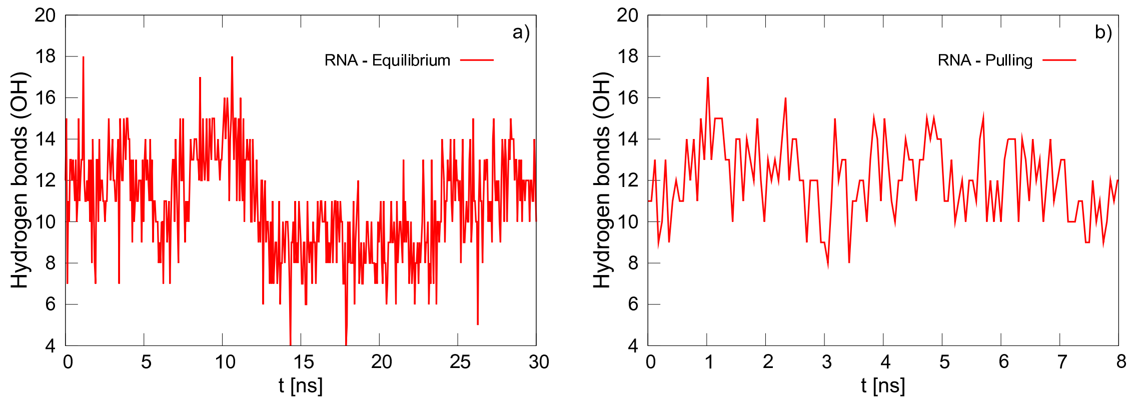

FIG. S5: **Pulling Simulations**. Number of intramolecular hydrogen bonds formed by the OH group of the ribose sugar of RNA quadruplex.

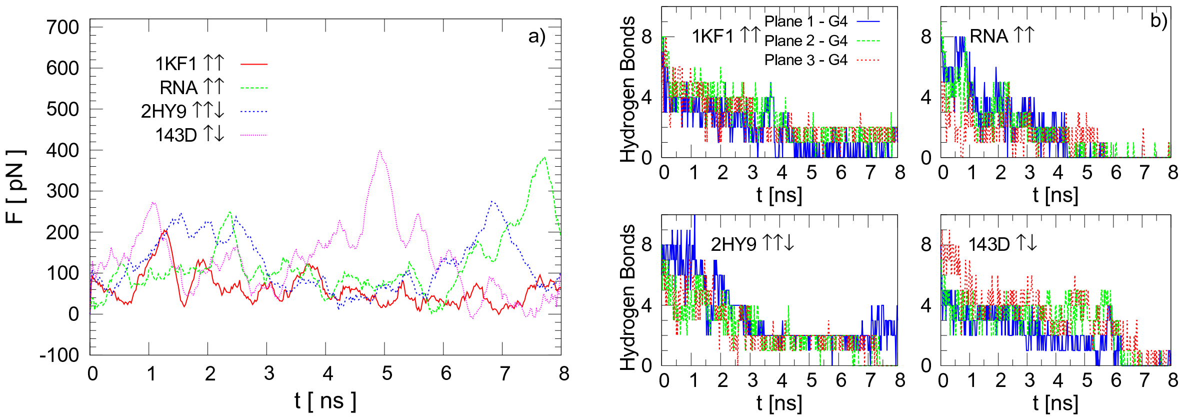

FIG. S6: **Pulling Simulations**. Pulling simulations without the central ions. a) Forces as a function of the time. b) Number of hydrogen bonds in each plane.

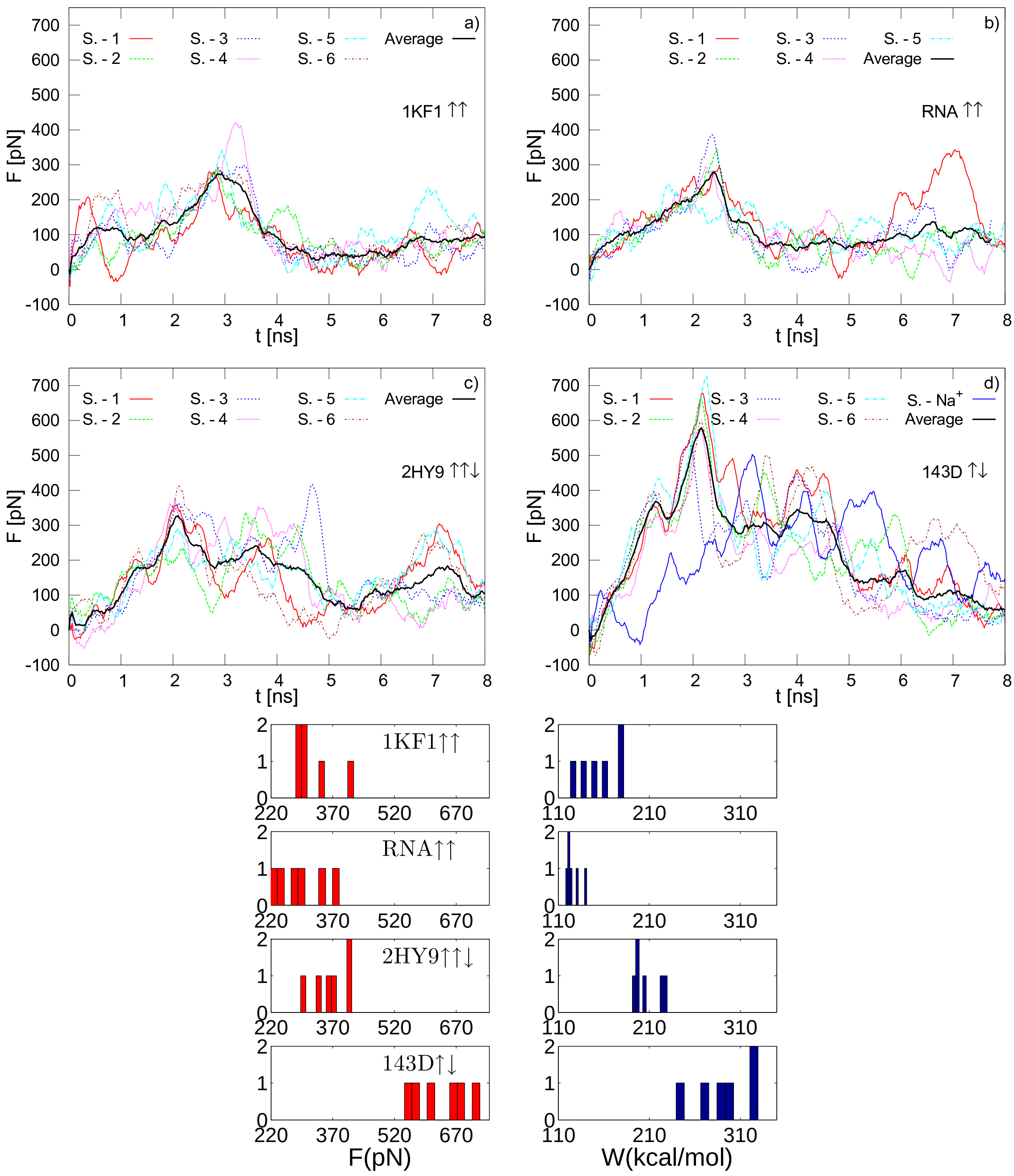

FIG. S7: **Pulling Simulations**. Smooth force curves for different realizations of the pulling. a) 1KF1 $\uparrow\uparrow$ (Parallel). b) RNA $\uparrow\uparrow$ (Parallel). c) 2HY9 $\uparrow\uparrow\downarrow$ (Hybrid). d) 143D $\uparrow\downarrow$ (Antiparallel). For the antiparallel quadruplex the force curve of one simulation with Na^+ ions has also been included. The eight bottom panels show the distribution of the maximum force (left panels, red) and the work (right panels, blue) for each trajectory of every quadruplex. It is there visible that even if the maximum force is similar for the parallel and hybrid structures, the calculated unfolding energy appears different. In the order of lower to higher energy, we find: RNA $\uparrow\uparrow$, 1KF1 $\uparrow\uparrow$, 2HY9 $\uparrow\uparrow\downarrow$, and 143D $\uparrow\downarrow$. The antiparallel configuration remains in all the cases the most stable.

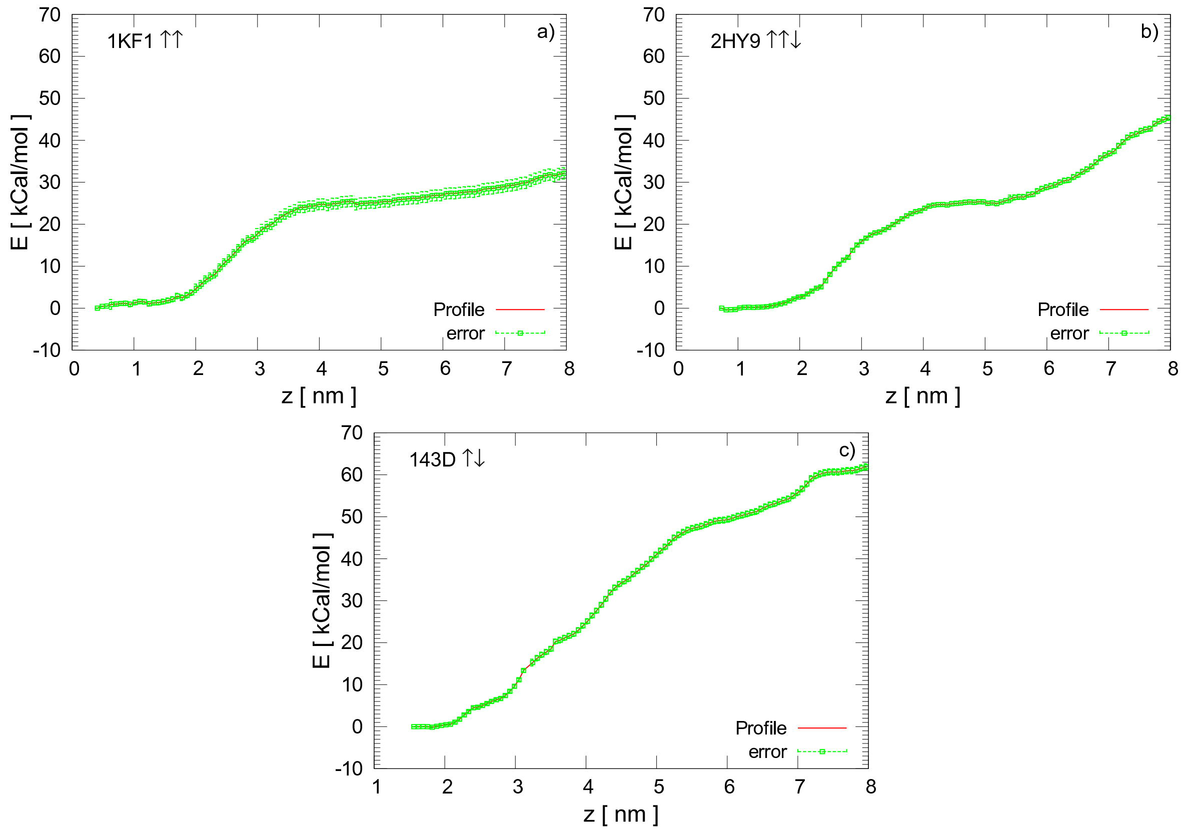

FIG. S8: **Potential of Mean Force**. PMF with the estimated error obtained from bootstrap analysis for the DNA intramolecular G-quadruplexes. a) Parallel 1KF1 ↑↑. b) Hybrid 2HY9 ↑↑. c) Antiparallel 143D ↑↓.

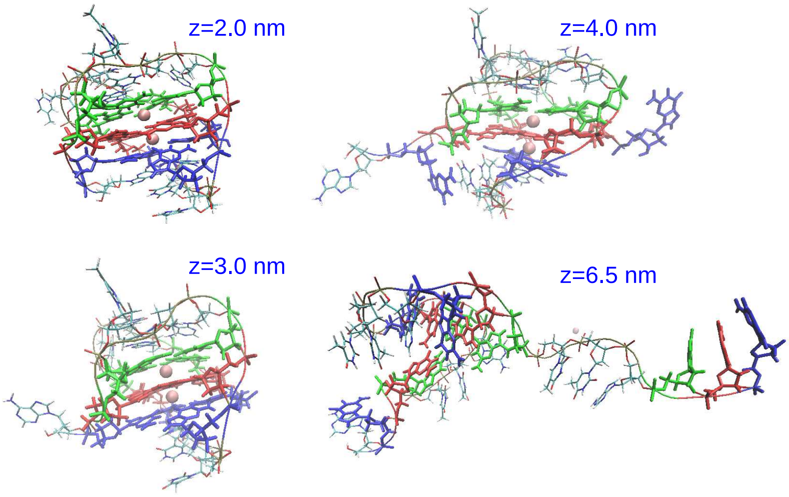

FIG. S9: **Potential of Mean Force**. Snapshots from the equilibrium simulations at different extensions z for the 143D ↑↓ quadruplex.

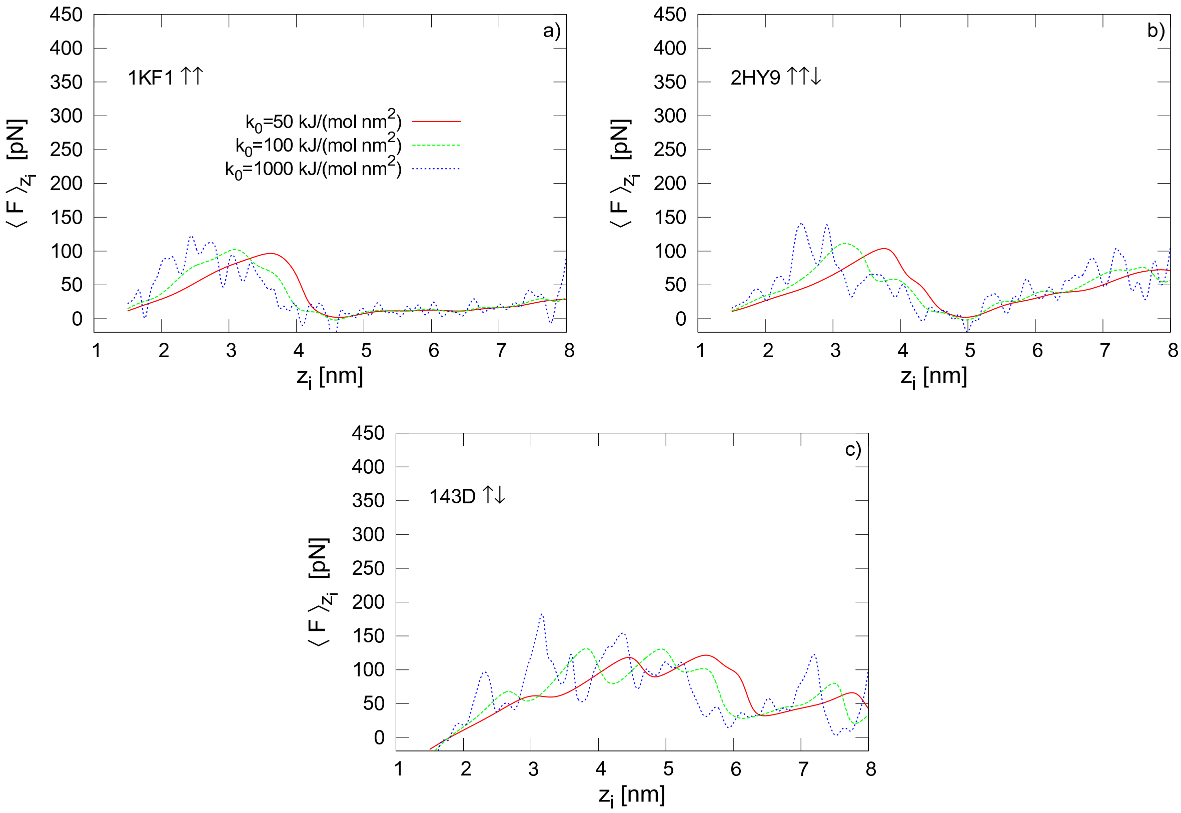

FIG. S10: **Potential of Mean Force**. Average equilibrium force extension curves at different values of the elastic constant k_0 for the DNA intramolecular G-quadruplexes. These curves are derived from the PMF by using the equation 1.
